# Insomnia disorders are associated with increased cardiometabolic disturbances and death risks from cardiovascular diseases in psychiatric patients treated with weight-gain-inducing psychotropic drugs: results from a Swiss cohort

**DOI:** 10.1186/s12888-022-03983-3

**Published:** 2022-05-17

**Authors:** Nermine Laaboub, Céline Dubath, Setareh Ranjbar, Guibet Sibailly, Claire Grosu, Marianna Piras, Didier Délessert, Hélène Richard-Lepouriel, Nicolas Ansermot, Severine Crettol, Frederik Vandenberghe, Carole Grandjean, Aurélie Delacrétaz, Franziska Gamma, Kerstin Jessica Plessen, Armin von Gunten, Philippe Conus, Chin B. Eap

**Affiliations:** 1 Unit of Pharmacogenetics and Clinical Psychopharmacology, Department of Psychiatry, Centre for Psychiatric Neuroscience, Lausanne University Hospital, University of Lausanne, 1008 Prilly, Prilly, Switzerland; 2 Center for Psychiatric Epidemiology and Psychopathology, Department of Psychiatry, Lausanne University Hospital, University of Lausanne, Prilly, Switzerland; 3 Prison Medicine and Psychiatry Service, Department of Psychiatry, Lausanne University Hospital, University of Lausanne, Prilly, Switzerland; 4 Unit of Mood Disorders, Department of Psychiatry, Geneva University Hospital, Geneva, Switzerland; 5 Les Toises Psychiatry and Psychotherapy Center, Lausanne, Switzerland; 6 Service of Child and Adolescent Psychiatry, Department of Psychiatry, Lausanne University Hospital, University of Lausanne, Prilly, Switzerland; 7 Service of Old Age Psychiatry, Department of Psychiatry, Lausanne University Hospital, University of Lausanne, Prilly, Switzerland; 8 Service of General Psychiatry, Department of Psychiatry, Lausanne University Hospital, University of Lausanne, Prilly, Switzerland; 9 School of Pharmaceutical Sciences, University of Geneva, University of Lausanne, Geneva, Switzerland; 10 Center for Research and Innovation in Clinical Pharmaceutical Sciences, University of Lausanne, Lausanne, Switzerland; 11 Institute of Pharmaceutical Sciences of Western Switzerland, University of Geneva, University of Lausanne, Lausanne, Switzerland

**Keywords:** Psychiatry, Insomnia disorders, Metabolic syndrome, Metabolic worsening, Cardiovascular diseases

## Abstract

**Study objectives:**

Insomnia disorders as well as cardiometabolic disorders are highly prevalent in the psychiatric population compared to the general population. We aimed to investigate their association and evolution over time in a Swiss psychiatric cohort.

**Methods:**

Data for 2861 patients (8954 observations) were obtained from two prospective cohorts (PsyMetab and PsyClin) with metabolic parameters monitored routinely during psychotropic treatment. Insomnia disorders were based on the presence of ICD-10 “F51.0" diagnosis (non-organic insomnia), the prescription of sedatives before bedtime or the discharge letter. Metabolic syndrome was defined using the International Diabetes Federation definition, while the 10-year risk of cardiovascular event or death was assessed using the Framingham Risk Score and the Systematic Coronary Risk Estimation, respectively.

**Results:**

Insomnia disorders were observed in 30% of the cohort, who were older, predominantly female, used more psychotropic drugs carrying risk of high weight gain (olanzapine, clozapine, valproate) and were more prone to suffer from schizoaffective or bipolar disorders. Multivariate analyses showed that patients with high body mass index (OR = 2.02, 95%CI [1.51–2.72] for each ten-kg/m^2^ increase), central obesity (OR = 2.20, [1.63–2.96]), hypertension (OR = 1.86, [1.23–2.81]), hyperglycemia (OR = 3.70, [2.16–6.33]), high density lipoprotein hypocholesterolemia in women (OR = 1.51, [1.17–1.95]), metabolic syndrome (OR = 1.84, [1.16–2.92]) and higher 10-year risk of death from cardiovascular diseases (OR = 1.34, [1.17–1.53]) were more likely to have insomnia disorders. Time and insomnia disorders were associated with a deterioration of cardiometabolic parameters.

**Conclusions:**

Insomnia disorders are significantly associated with metabolic worsening and risk of death from cardiovascular diseases in psychiatric patients.

**Supplementary Information:**

The online version contains supplementary material available at 10.1186/s12888-022-03983-3.

## Introduction

Prevalence rates of metabolic syndrome (MetS) and cardiovascular diseases (CVD) are higher in psychiatric patients compared to the general population [1, 2], with many contributing factors including the psychiatric illness [3], lifestyle (e.g., unhealthy diet, lack of physical exercise, smoking, excessive alcohol use) [4], clinical and genetic factors [5]. Hence, life expectancy of psychiatric patients is reduced by over 10 years with a mortality rate of two to four times higher than the general population [6, 7]. Sleep disorders are a common complaint at all ages, in the general as well as in the psychiatric populations and are, therefore, a major public health problem. In the general population, prevalence rates of sleep disorders range from 35 to 52% [8] while up to 90% of depressed patients are reporting sleep disorders [9]. Insomnia, which symptoms are difficulty initiating or maintaining sleep and/or non-restorative sleep, is the most prevalent sleep disorder [10], particularly in patients suffering from depression, generalized anxiety and post-traumatic stress disorders [9]. In fact, the co-occurrence of psychiatric and insomnia disorders can drastically affect patients’ safety by increasing the risk of relapse of their psychiatric symptoms and even suicide [11, 12]. Nevertheless, although insomnia disorders may negatively affect psychiatric patients, they are poorly taken into account and often not reported in medical files. Thus, caregivers may focus on managing the main psychiatric diagnosis, prescribing sedative medications for sleep disorders namely insomnia and abstaining from more in-depth investigation into the causes. However, psychotropic medications might exacerbate or even induce sleep disorders, with many antipsychotics causing restless leg syndrome, periodic limb movements and/or weight gain, which are associated with sleep disorders [13].

Sleep disorders as well as psychiatric disorders are thus associated with increased metabolic disturbances including obesity [14, 15], diabetes [16, 17], hypertension [18, 19], dyslipidemia [1, 20] and MetS [1, 21] leading to cardiovascular diseases and thus increasing risk of premature death [2, 22].

Many epidemiological studies of sleep disorders and metabolic disturbances have been conducted in the general population as well as in psychiatric cohorts [23, 24]. Evidence concerning the association between sleep-related breathing disorders (i.e. respiratory disorders occurring during sleep) and metabolic disturbances is convincing [25]; however, that is not the case when considering non-organic sleep disorders (i.e., non-organic insomnia) [25–27]. Of note, a recent population based cohort study revealed higher hazard of all-cause mortality in participants with insomnia symptoms (i.e., difficulty initiating sleep, difficulty maintaining sleep, early-morning awakening, and non-restorative sleep) as compared to those who were symptom-free [28]. On the other hand, only a few studies with small sample sizes have so far focused on non-organic sleep disorders in the psychiatric population [20, 24, 29] and, to our knowledge, no study specifically included patients taking weight-gain-inducing psychotropic drugs; these patients are at high risk for cardiometabolic disturbances. Moreover, studies have used a single measure of sleep, as well as cardiometabolic parameters, whereas both may change over time, especially in this psychiatric population.

We therefore sought, first, to investigate the association between insomnia disorders and cardiometabolic disturbances (MetS and/or its five components, namely central obesity, hypertension, hyperglycemia, hypertriglyceridemia, and high-density lipoprotein (HDL) hypocholesterolemia; body mass index (BMI) and 10-year CVD risk) in a Swiss psychiatric cohort taking weight-gain-inducing psychotropic drugs. Second, we aimed to look at the evolution of cardiometabolic parameters and their associations with insomnia disorders over time.

## Methods

### Study design, setting and participants

The Department of Psychiatry of the University Hospital of Lausanne has established a guideline for the follow-up of patients starting or already receiving psychotropic treatment known to present a potential risk of inducing metabolic disorders (listed in Supplementary Table 1). Thus, samples are collected during routine clinical examinations with various information on physical health risk factors such as BMI, waist circumference (WC), fasting plasma glucose (FPG), lipid profile, blood pressure (BP) and smoking. Taking advantage of this guideline, a longitudinal observational study (PsyMetab), approved by the ethics committee of the canton of Vaud (CER-VD) has been underway since 2007, recruiting patients who have given written informed consent [30]. In addition, due to the non-interventional post-hoc analysis study design, the CER-VD approved the use of clinical data of followed-up patients in the Department of Psychiatry of the Lausanne University Hospital between 2007 and 2015 without informed consent (PsyClin). Observations collected between January 1, 2007, and August 26, 2020, were included.

### Insomnia disorders

The protocol for defining insomnia disorders has been published previously [30]. Indeed, insomnia disorders were defined by the presence of at least one of the following criteria: for inpatients, the ICD-10 “F51.0" diagnosis (non-organic insomnia), if available in medical files or the prescription of sedative drugs before bedtime, taking into account the dosage and the prescription condition (prescription for insomnia, Supplementary Table 2). When these two variables were not available, or because some sedative drugs could be prescribed for medical reasons other than insomnia disorders, the entire discharge letter of the corresponding hospital stay was screened to detect or confirm the presence of insomnia disorders, including difficulty in initiating or maintaining sleep, insomnia or repeated nocturnal awakenings. For outpatients, those receiving the sedative drugs melatonin, zolpidem, zopiclone, or herbal sedatives (valerian and hops) were considered to have insomnia disorders and those not receiving such drugs were considered not to have insomnia disorders. Outpatients receiving the other sedative drugs listed in Supplementary Table 2 were excluded as information on the time of drug intake and dosage, as well as clinical reports, were not available in the electronic files.

### Cardiometabolic disturbances

Metabolic parameters including weight (kg), height (cm), WC (cm), systolic and diastolic BP (mmHg), FPG (mmol/L), triglycerides (TG; mmol/L), HDL cholesterol (HDL-C; mmol/L), low-density lipoprotein cholesterol (LDL-C; mmol/L) and total cholesterol (total-C; mmol/L) were obtained from patient medical files.

Metabolic disturbances were defined as summarized in Supplementary Table 3 and MetS was defined using the International Diabetes Federation (IDF) definition [31].

CVD risk was estimated using the Framingham Risk Score (FRS) [32] and the Systematic Coronary Risk Estimation (SCORE) [33], which predict the risk of developing or dying from CVD within ten years, respectively, both adapted to the Swiss population [34, 35].

### Statistical analyses

Descriptive data were shown as the number of observations and percentages for categorical variables or weighted mean and standard deviation (SD) for continuous ones. We compared groups of patients with and without insomnia disorders using Pearson Chi-squared tests or t-tests considering the hierarchical structure in the data for categorical and continuous variables, respectively. To investigate the association between insomnia disorders and cardiometabolic disturbances (MetS and its five components, BMI and CVD risk), generalized linear mixed-effect models characterized by penalizing quasi-likelihood were used. Multivariate models were adjusted for age, sex, smoking status and psychotropic medication (classified according to weight-gain risk) based on a priori knowledge of their association with insomnia disorders and/or cardiometabolic disturbances. Models adjusted for psychiatric diagnosis were also made. As 28% of data were missing the psychiatric diagnosis and the results were almost identical for the variables of interest, this covariate was therefore excluded from the analysis. The models for CVD risk estimations were adjusted for psychotropic medication.

In addition, linear mixed-effect models were used to examine the association between cardiometabolic parameters (BMI, WC, BP, FPG, TG, HDL-C, LDL-C, Total-C and CVD risks [FRS and SCORE]) and insomnia disorders adjusting for time, which was defined as the duration between each two consecutive observations. In order to ensure a normal distribution of variables, TG and CVD risk variables were log-transformed and time was scaled to have a mean of 0 and SD of 1.

Data preparation and univariate analyses were conducted using Stata 16.1 (StataCorp; College Station, Texas), multivariate analyses were performed using the R environment for statistical computing version 4.0.2 and correction for multiple testing was applied using the false discovery rate.

## Results

The cohort characteristics of the 2861 included patients (8954 observations, Supplementary Fig. 1) are shown in Table 1.

**Table 1:** Demographic and clinical characteristics

	**Without insomnia disorders (*N* = 6290; 70%)**	**With insomnia disorders (*N* = 2664; 30%)**	***P*-value**
**Age in years**, Wmean (SD)	39 (16)	45 (17)	** < 10**^**–4**^
**Gender** (women), N (%)	2921 (46)	1411 (53)	** < 10**^**−***3*^
**Smokers (yes)**, N (%)	2976 (53)	1350 (55)	0.06
**Psychiatric diagnosis **^a^ N (%)**:**			** < 10**^**–3**^
Others	743 (17)	324 (16)	
Psychotic disorders	1641 (37)	625 (31)	
Schizoaffective disorders	359 (8)	264 (13)	
Bipolar disorders	753 (17)	390 (20)	
Depression	934 (21)	399 (20)	
**Risk of weight gain for psychotropic drugs **^b^**,** N (%):			** < 10**^**–3**^
Low risk	1307 (21)	457 (17)	
Medium risk	3751 (59)	1569 (59)	
High risk	1230 (20)	638 (24)	
**BMI** kg/m^2^, Wmean (SD)	25.5 (5.4)	25.9 (5.7)	**0.001**
**WC** cm, Wmean (SD)	91 (15)	93 (16)	** < 10**^**–4**^
**BP** mmHG, Wmean (SD):			
Systolic	122 (16)	122 (16)	0.82
Diastolic	79 (12)	79 (12)	0.56
**FPG** mmol/L, Wmean (SD)	5.03 (1.15)	5.22 (1.32)	** < 10**^**–4**^
**Plasma cholesterol**, mmol/L, Wmean (SD):			
HDL	1.36 (0.41)	1.37 (0.43)	0.47
LDL	2.80 (0.93)	2.86 (0.93)	0.08
Triglycerides	1.44 (1.00)	1.41 (0.88)	0.31
Total	4.83 (1.10)	4.87 (1.11)	0.23

Analyses were conducted using chi2 and t-tests for clustered data. Significant P-value in bold

^a^Diagnoses were based on the ICD-10 classification, and were classified as: others [F00-F19; F34-F99] | psychotic disorders [F20-F24; F28-F29] | schizoaffective disorders [F25] | bipolar disorders [F30-F31] | depression [F32-F33]

^b^Psychotropic drugs were classified according to the risk of weight gain as follows: low risk: amisulpride, aripiprazole, chlorprothixene, flupentixol, haloperidol, lurasidone, sulpiride, tiapride, brexpiprazole; medium risk: asenapine, carbamazepine, amitriptyline, clomipramine, levomepromazine, lithium, mirtazapine, paliperidone, quetiapine, risperidone, trimipramine, zuclopenthixol; high risk: clozapine, olanzapine, valproate. Abbreviations: *BMI* body mass index, *BP* blood pressure, *cm* centimeter, *FPG* fasting plasma glucose, *HDL* high-density lipoprotein, *kg/m*^*2*^ kilogram per square meter, *LDL* low-density lipoprotein, *mmHG* millimeters of mercury, *mmol/L* millimoles per liter, *N* number, *SD* standard deviation, *WC* waist circumference, *Wmean* weighted mean

Insomnia disorders were observed in 30% of the cohort (*N* = 2664 observations), who were older (weighted mean age 45 vs 39 years, *p* < 10^–4^), predominantly female (53% vs 46%, *p* < 10^–3^), more prone to suffer from schizoaffective and bipolar disorders (13% vs 8% and 20% vs 17%), and less prone to suffer from psychotic disorders (31% vs 37%, *p* < 10^–3^). Psychotropic drugs inducing high risk of weight gain were more often prescribed in patients with insomnia disorders (24% vs 20%), while those with low risk of weight gain were less often prescribed in such patients (17% vs 21%, *p* < 10^–3^). A trend toward a higher proportion of smokers was observed in the group with insomnia disorders (55% vs 53%, *p* = 0.06).

Patients with insomnia disorders had higher BMI (weighted mean: 25.9 vs 25.5 kg/m^2^, *p* = 0.001), WC (weighted mean: 93 vs 91 cm, *p* = 10^–4^), FPG (weighted mean: 5.22 vs 5.03 mmol/L, *p* < 10^–4^), and a trend for higher LDL-C (weighted mean: 2.86 vs 2.80 mmol/L, *p* = 0.08, see Table 1). No significant differences were observed for systolic and diastolic BP, HDL-C, TG and Total-C plasma levels.

Prevalence rates of central obesity, hypertension, hyperglycemia, HDL hypocholesterolemia and MetS were higher in patients with insomnia disorders (58% vs 52%; *p* < 10^–4^, 54% vs 43%; *p* < 10^–4^, 30% vs 20%; *p* < 10^–4^, 38% vs 34%; *p* = 0.0003 and 25% vs 17%; *p* < 10^–4^, respectively, Table 2). No significant difference was observed for hypertriglyceridemia.

**Table 2:** Prevalence of metabolic disturbances according to the IDF definition and estimation of 10-year risk of cardiovascular disease

	**Without insomnia disorders (*N* = 6290; 70%)**	**With insomnia disorders (*N* = 2664; 30%)**	***P*-value**
**A. Central Obesity,** N (%)	3233 (52)	1532 (58)	** < 10**^**–4**^
**B. Hypertension**, N (%)	1947 (43)	991 (54)	** < 10**^**–4**^
**C. Hyperglycemia**, N (%)	635 (20)	421 (30)	** < 10**^**–4**^
**D. HDL Hypocholesterolemia**, N (%)	1175 (34)	656 (38)	**0.0003**
**E. Hypertriglyceridemia**, N (%)	1081 (31)	548 (33)	0.33
**F. Metabolic syndrome,** N (%)	795 (17)	477 (25)	** < 10**^**–4**^
**G. FRS (%)**, Wmean (SD)	1.58 (3.07)	1.84 (3.26)	**0.04**
**Prevalence **^**a**^, N (%):			0.10
Very low risk	2343 (95)	871 (93)	
Low risk	69 (3)	41 (4)	
Intermediate risk	50 (2)	18 (2)	
High risk	15 (< 1)	8 (< 1)	
**H. SCORE (%),** Wmean (SD)	0.57 (1.66)	0.93 (2.13)	** < 10**^**–4**^
**Prevalence** ^b^, N (%):			** < 10**^**−4**^
Very low risk	2212 (91)	798 (85)	
Low risk	105 (4)	40 (4)	
Intermediate risk	68 (3)	50 (6)	
High risk	54 (2)	48 (5)	

Analyses were conducted using chi2 and t-tests for clustered data. Significant P-value in bold

A. Defined using the IDF definition as follows: waist circumference: men ≥ 94 cm; women ≥ 80 cm; or BMI > 30 kg/m^2^

B. Defined as follows: systolic BP ≥ 130 or diastolic BP ≥ 85 mm Hg or treatment for hypertension

C. Defined as follows: fasting plasma glucose ≥ 5.6 mmol/L or treatment for type 2 diabetes

D. Defined as follows: HDL cholesterol: men < 1.03 mmol/L; women < 1.29 mmol/L or treatment for lipid abnormality

E. Defined as follows: triglycerides ≥ 1.7 mmol/L or treatment for lipid abnormality

F. Defined using the IDF definition as follows: presence of the A factor plus any two of the following: B / C / D and/or E factors

G. Estimated risk of developing cardiovascular disease within 10 years

H. Estimated risk of death from cardiovascular disease within 10 years. Both scores were adapted to the Swiss population

^a^ FRS prevalence risk score categories were defined as follows: very low risk (< 6%); low risk (6–10%); intermediate risk (10–20%) and high risk (> 20%)

^b^ SCORE prevalence risk categories were defined as follows: very low risk (< 1.5%); low risk (≥ 1.5% & < 2.5%); intermediate risk (≥ 2.5% & < 5%); high risk (> 5%). Abbreviations: *FRS* Framingham Risk Score, *HDL* high-density lipoprotein, *IDF* International Diabetes Federation, *N* number, *SCORE* Systematic Coronary Risk Estimation, *SD* standard deviation, *Wmean* weighted mean

In addition, the 10-year risk of developing or dying from CVD was higher in patients with insomnia disorders (weighted mean FRS: 1.84% vs 1.58%; *p* = 0.04 and weighted mean SCORE: 0.93% vs 0.57%; *p* < 10^–4^, respectively, Table 2) as compared to those without insomnia disorders. Moreover, the percentage of patients with a higher 10-year risk of dying from CVD (SCORE > 5% [1]) was greater in patients with insomnia disorders, whereas the percentage of patients with very low risk (SCORE < 1.5% [1]) was lower, compared to those without insomnia disorders (5% vs 2% and 85% vs 91%, *p* < 10^–4^, respectively, see Table 2).

Multivariate analyses (Supplementary Table 4) showed that age, female sex, smoking and the use of medium- or high-risk weight-gain-inducing psychotropic drugs were associated with the occurrence of insomnia disorders. With each ten-year age increase, insomnia disorder risk was doubled (OR=1.99-2.26, *p*<10^-3^, depending on the model used). Women were about two times more at risk of insomnia disorders (OR=1.89–2.79, p≤0.002), and smokers were up to three times more at risk (OR=2.29–3.01, *p*≤0.001,). Psychotropic drugs with medium or high risk of weight gain roughly doubled the risk of insomnia disorders (OR_medium_=1.82–2.10, *p*≤0.006; OR_high_=2.38-2.99, *p*≤0.02).

**Fig. 1:**
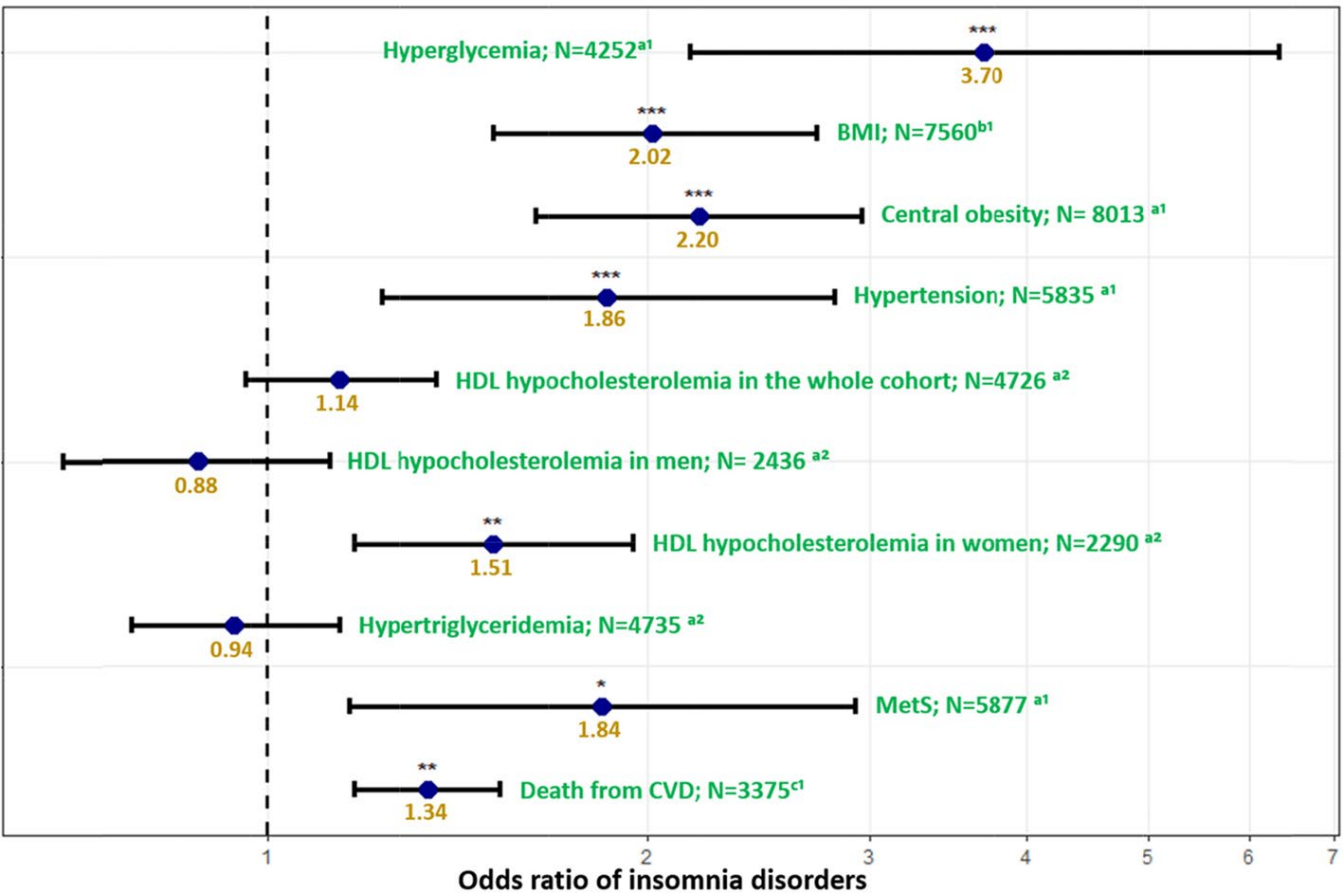
^a^Defined using the International Diabetes Fedearation definition; ^b^BMI by 10kg.m-^2^. ^c^Estimated risk of death from cardiovascular disease within 10 years using the Systematic Coronary Risk Estimation. Models were adjusted for age, sex, smoking status, and pyschotropic medication (classified by the risk of weight gain), except the model for CVD was adjusted only for pyschotropic medication. ^1^Models fitted with random effect at observation level. ^2^Models fitted with random effect at patient level. ***:*p*-value < 0.001; **:*p*-value ≤ 0.01. Correction for multiple testing was applied using false discovery rate. Abbreviations: BMI body mas index, CVD cardiovascular diseases, HDL high-density lipoprotein, MetS metabolic syndrome, *N* number

With respect to metabolic disturbances, the highest association was observed with hyperglycemia. Thus, patients suffering from hyperglycemia were more than three and a half times more at risk of insomnia disorders (OR = 3.70, 95% CI [2.16–6.33]; Fig. 1 and Supplementary Table 4). In addition, each ten-kg/m^2^ increase of BMI was shown to double this risk (OR = 2.02, 95% CI [1.51–2.72]; Fig. 1 and Supplementary Table 4). Moreover, those suffering from central obesity, hypertension and MetS were more prone to have insomnia disorders (OR_central obesity_ = 2.20, 95% CI _central obesity_ [1.63–2.96]; OR_hypertension_ = 1.86, 95% CI _hypertension_ [1.23–2.81]); OR_MetS_ = 1.84, 95% CI _MetS_ [1.16–2.92]). The association between HDL hypocholesterolemia and insomnia disorders in the whole sample was not statistically significant (Fig. 1). However, the association between insomnia disorders and sex was significant (OR = 1.70, *p* = 0.003); stratified analysis by sex showed that women with HDL hypocholesterolemia were 51% more prone to have insomnia disorders (OR = 1.51, 95% CI [1.17–1.95]). We did not find a significant association among men (Fig. 1). No association was found between hypertriglyceridemia and insomnia disorders (Fig. 1). Finally, a one-unit increase in our score measuring the risk of death from CVD within 10 years increased the risk of insomnia disorders by 34% (OR = 1.34, 95% CI [1.17–1.53]; Fig. 1 and Supplementary Table 4).

Expectedly, time was significantly associated with an increase in BMI, WC and FPG levels, with a trend toward increased LDL-C levels, while HDL-C levels decreased over time in women. Regarding the 10-year CVD risk, a trend toward decreased risk of developing CVD over time was observed, while the decrease in risk of death from CVD was statistically significant (Supplementary Table 5).

**Fig. 2:**
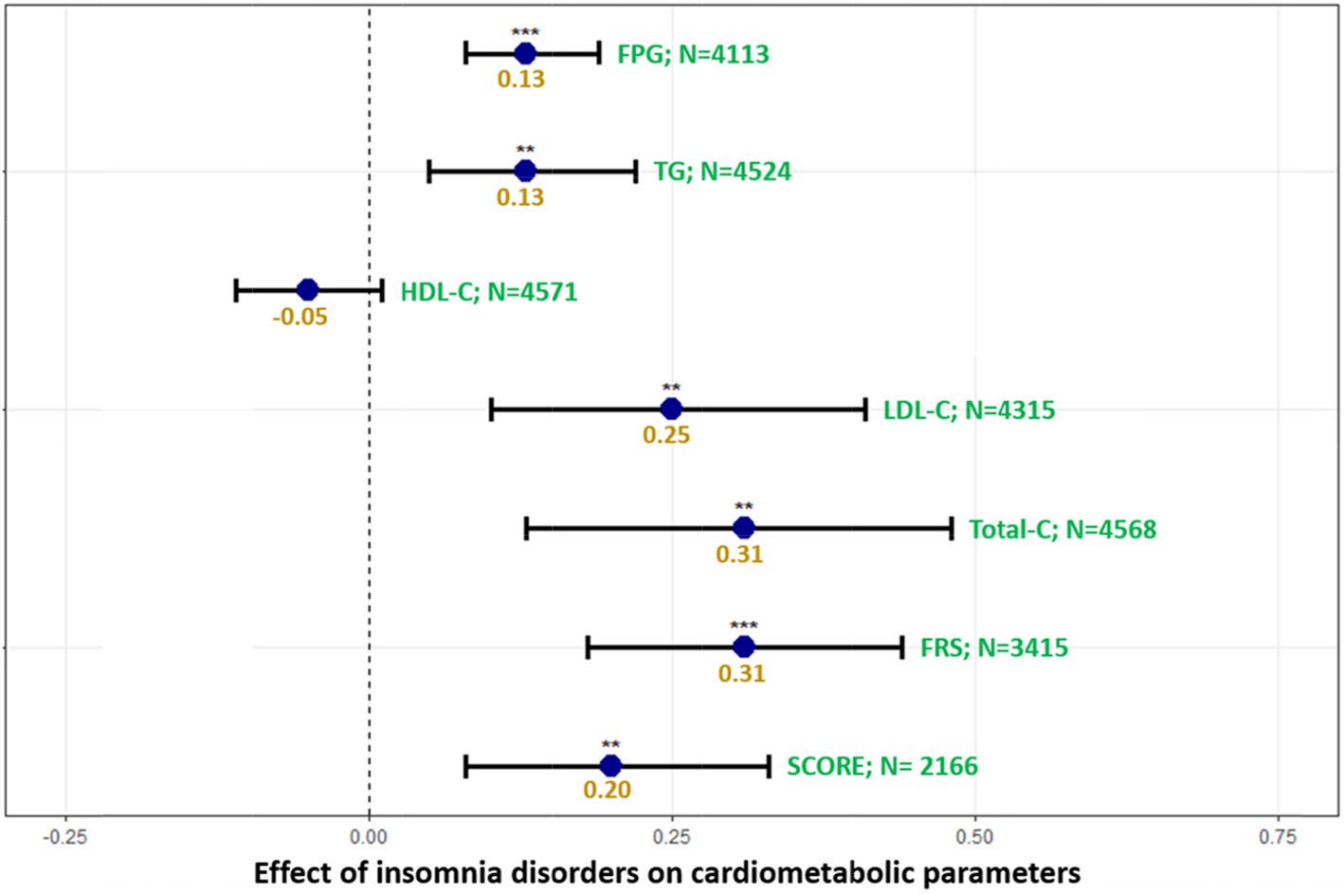
Model for FPG was adjusted for time,age, sex, smoking status and pyschotropic medication. Model for TG was adjusted for time, age, interaction betwwen age and imsonia disorders, sex, smoikng status, setting of care (in/outpatient) and pyschotropic medication. Model for HDL-C was adjusted for time, age, interaction between age and imsonia disorders, smoking status and pyschotropic medication. Model for LDL-C was adjusted for time, age, interaction between age and imsonia disorders, sex and smoking status. Model for Total-C was adjusted for time, age, sex, interaction between age and imsonia disorders, sex, smoking status and pyschotropic medication. Models for 10-year CVD risks (FRS and SCORE) were adjusted for time and pyschotropic medication. ***:*p*-value < 0.001; **p-value < 0.01; *:*p*-value ≤ 0.05. Correction for multiple was applied using false discovery rate. Abbreviations: FPG fasting plasma glucose, FRS Framingham Risk Score, HDL-C high-density lipoprotein cholesterol, LDL-C low-density lipoprotein cholesterol, N number, SCORE  Systematic Coronary Risk Estimation, Total-C total cholesterol, TG triglycerides

**Fig. 3:**
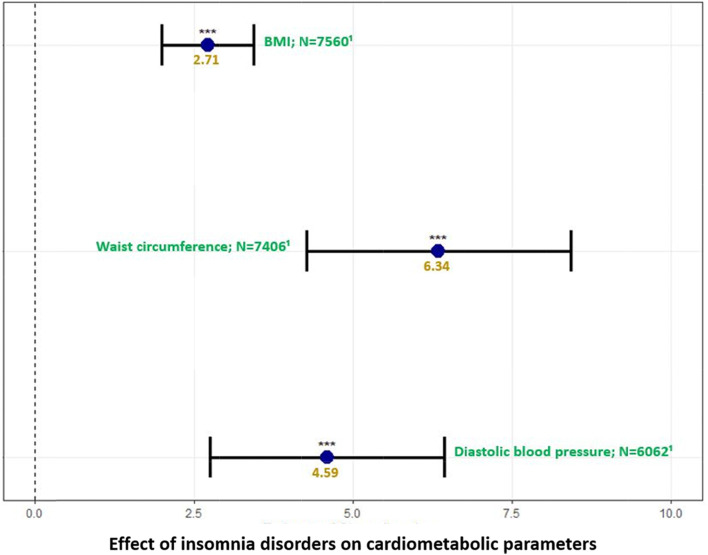
Models for BMI and waist circumference were adjusted for time, age, interaction between age and sleep disorders, sex, smoking status, and psychotropic medication. Model for diastolic blood pressure was adjusted for time, age, interaction between age and sleep disorders, sex and psychotropic medication. ***:*p*-value < 0.001; **:*p*-value < 0.01;*:*p*-value ≤ 0.05. Correction for multiple testing was applied using false discovery rate. Abbreviations: BMI body mas index, N  number

In addition, patients with insomnia disorders had an average increased BMI of 2.71 [1.99–3.44] kg/m^2^, WC of 6.34 [4.27–8.42] cm, diastolic BP of 4.59 [2.75–6.43] mmHG, TG of 0.13 [0.05–0.22] mmol/L, LDL-C of 0.25 [0.10–0.41] mmol/L, total-C of 0.31 [0.13–0.48] mmol/L; however, all these effects decreased with age (Figs. 2 and 3 and Supplementary Table 5). Moreover, patients with insomnia disorders had an increased FPG of 0.13 [0.08–0.19] mmol/L, along with 10-year risks of developing or dying from CVD of 31% [18%-44%] and 20% [8%-33%], respectively, (Fig. 2 and Supplementary Table 5). No significant decrease in HDL-C was observed in those with insomnia disorders after adjusting for time spent between each two consecutive observations, either in the whole cohort or when considering women only (Fig. 2 and Supplementary Table 5).

## Discussion

In the present study, 2861 patients (8954 observations) were included, of which 30% had insomnia disorders, which is in line with previous epidemiological studies in this population [36]. To our knowledge, the present study is the first showing in a very high-risk group, namely psychiatric patients taking weight-gain-inducing psychotropic drugs, the association between insomnia disorders and cardiometabolic disturbances including higher BMI, central obesity, hypertension, hyperglycemia, HDL hypocholesterolemia, MetS and the 10-year risks of developing or dying from CVD, also considering the deterioration of metabolic parameters over time when suffering from insomnia disorders.

The most significant association was observed between insomnia disorders and hyperglycemia. Thus, patients with hyperglycemia were more than three and a half times more likely to have insomnia disorders. In the literature, this association is inconsistent due to the heterogeneity of this sleep disorders and hyperglycemia classification (e.g., several studies used diabetes mellitus whereas others used hyperglycemia for their classification [16, 27]). Nevertheless, insomnia disorders affect neuroendocrine system activity by increasing sympathetic nerve activity, which decreases β-cell responsiveness and increases cortisol secretion leading to insulin reduction and resistance, respectively [37, 38]. Furthermore, when associated with decreased leptin and increased ghrelin levels, which increase the sense of hunger and appetite and calorie intake [39], insomnia disorders lead to longer eating time, increased fatigue and lower physical activity resulting in, among other consequences, weight gain and diabetes. Moreover, insomnia disorders could induce melanin-concentrating hormone dysregulation, which is involved in leptin and ghrelin levels, in sleep, eating-habit and energy metabolism regulation and stress responses, which in turn increase insulin resistance [40]. Finally, compared to the general population, psychiatric patients are more often prescribed statins, which can lead to increased insulin resistance [1]. Conversely, psychiatric patients taking psychotropic medication, especially atypical antipsychotics, are more prone to suffer from diabetes mellitus [1, 13], of which the complications (polyuria, nocturia, polydipsia, diabetic neuropathy inducing restless leg syndrome and diabetic retinopathy) are associated with insomnia disorders [41].

There is a well-documented association between higher BMI and sleep disorders, especially obstructive sleep apnea (a disorder characterized by frequent stops of breathing during sleep) [42], but not with non-organic sleep disorders namely insomnia. An 18% increased risk of obesity has been shown, while other studies reported no such association [25–27]. In the present study, an increase of BMI by 10 kg/m^2^ is associated with double the risk of insomnia disorders. Thus, higher BMI in patients with insomnia disorders could, at least in part, be explained by an increased sense of hunger/appetite and increased eating time [39]. Furthermore, preference for an unhealthy diet mainly characterized by high fat and carbohydrate intake, can also increase BMI and insomnia disorders onset by increasing body temperature and heart rate and reducing nocturnal secretion of melatonin [14]. As mentioned above, in addition to its association with increased food quantity and decreased food quality, insomnia disorders induce fatigue and daytime sleepiness leading to less physical exercise and energy expenditure, especially in psychiatric patients, who are already more sedentary than the general population [4]. Other potentially important mechanisms relating insomnia disorders to higher BMI and central obesity are stress and anxiety, which are known to be associated with insomnia disorders and are widely prevalent in psychiatric patients. In fact, adrenaline and then cortisol secretion are increased in order to cope with heightened energy metabolism required to respond to stressful conditions. As cortisol is secreted by the adrenal gland, fat could accumulate preferentially around the waist, to be metabolized quickly, leading to increased waist circumference. Thus, high cortisol concentrations are independently associated with risk of central obesity onset [43]. In fact, in the present study, psychiatric patients with insomnia disorders are two times more likely to have central obesity, a higher risk than the 20% increase reported in a general population cohort [44].

On the other hand, obesity can lead to insomnia disorders in psychiatric patients through several other mechanisms. First, psychotropic drugs, especially second-generation antipsychotics, are well known for their role in metabolic disturbances including weight gain [13]. High prevalences of obesity and central obesity were observed in the present cohort of patients treated with weight-gain-inducing psychotropic drugs. Indeed, multivariate analyses showed that patients with insomnia disorders were more likely to be treated with antipsychotics at medium or high risk of inducing weight gain. In addition, obesity is associated with several pathological conditions including respiratory diseases such as asthma, osteoarticular pains, and gastrointestinal disorders, which could induce insomnia disorders [14]. Finally, the pro-inflammatory cytokines (mainly present in visceral adipose tissue) are involved in sleep regulation and actually classified as “sleep-regulatory substances” [45, 46]. In fact, overweight and obese patients have the highest secretion of pro-inflammatory cytokines in the morning instead of during the night, which is associated with insomnia disorders [47].

In the present study, patients with hypertension were more prone to suffer from insomnia disorders, an association inconsistent across studies probably due to the heterogeneity of insomnia disorders and hypertension definitions [18, 25, 27]. However, short sleep durations are associated with an increase in stress hormones including adrenaline and cortisol, which increases blood pressure [48]. Moreover, hypertension often coexists with obesity and diabetes mellitus, which, as previously explained, are associated with insomnia disorders.

The association between insomnia disorders and dyslipidemia (e.g., elevated TG and lower HDL-C) has been the subject of several publications, raising a gender-specific difference in both dyslipidemia and insomnia disorder onset with significant association in women [20, 49]. In fact, sex hormones strongly affect lipoprotein metabolism and sleep [49, 50]. In the present study, after adjusting for covariates, insomnia disorders were neither associated with HDL hypocholesterolemia nor hypertriglyceridemia in the whole cohort. However, the interaction between HDL hypocholesterolemia and sex was significant when considering only women; those with HDL hypocholesterolemia were 51% more likely to have insomnia disorders but not hypertriglyceridemia.

Given the number and extent of associations between insomnia disorders and the above-mentioned metabolic disturbances, patients with MetS were expectedly 84% more at risk for insomnia disorders. In the general population, this association is inconsistent, with one study reporting no significant association [25] while another showed a 23% increased risk of metabolic syndrome in individuals with insomnia disorders [44], which is lower than the risk found in our psychiatric population. A study in major depressive patients showed an 80% increase in the risk of MetS in women (*N* = 433) but not in men nor in the whole cohort [24], while a recent study including 272 inpatients with severe psychiatric disorders reported a threefold higher risk [51]. To our knowledge, our study is the first to show such an association in a large psychiatric cohort.

A recent population based cohort study reported that respondents with one, two, three and four insomnia symptoms had a higher hazard risk of all-cause mortality compared to those who did not experience any insomnia symptoms [28]. The present study in a psychiatric cohort addressed the 10-year risk of death from CVD, and importantly, patients whose scores indicate high risk of death from CVD within 10 years were 34% more likely to have insomnia disorders. One meta-analysis reported that individuals from the general population with difficulties in initiating and maintaining sleep or the presence of nights of disturbed sleep were 45% more prone to have or to die from CVD as compared to good sleepers [22], while others have shown no significant association between short sleep and increased cardiovascular mortality [52]. Nevertheless, a recent study suggested a potential causal relationship showing that insomnia disorders increase risk of CVD [53]. For psychiatric patients, the association between insomnia disorders and morbidity/mortality due to CVD remained to be determined, although these patients are at increased risk of metabolic disturbances leading to CVD. To the best of our knowledge, the present study is the first to specifically assess this question in a large cohort of patients with severe mental illness. A deterioration of metabolic parameters was noted over time, which could be due to the use of psychotropic drugs with a risk of metabolic problems and to ageing [1, 54]. Paradoxically, our results show a decreased risk of developing or dying from CVD within 10 years over time which may be explained by the possible death or drop-out of some high-risk patients during the follow-up.

Several other risk factors for insomnia disorders were identified, including increasing age [55], sex (women) [50], and smoking [56], which are in agreement with previously published studies in general and psychiatric populations. Age is thus a strong predictor for insomnia disorders, since the quality and duration of sleep are altered with aging [55]. Women are more prone to suffer from insomnia disorders, both in general as well as psychiatric populations [50]. A higher proportion of women might suffer from stress, anxiety and/or depression, which can increase cortisol secretion and lead to insomnia disorders [57, 58].

The present study has several limitations. First, this is an observational longitudinal study and no causal relationship can be established. However, the results are in line with previous findings as well as bi-directional mechanisms linking insomnia disorders to worsening of metabolic parameters. Second, the classification of insomnia disorders was not determined by standard methods such as actigraphy or polysomnography which can provide information both on sleep quantity and quality. However, the use of these methods would be extremely difficult to implement in a large cohort of patients such as in the present study. In addition, the diagnostic classification of insomnia disorders as short-term or chronic one according to The International Classification of Sleep Disorders [59] could not be done. Furthermore, using the prescription of sedative medication to define insomnia disorders may increase the number of false negatives especially in outpatients. However, despite these restrictive criteria, associations between insomnia disorders and cardiometabolic disturbances were demonstrated. It must also be mentioned that a new version of the SCORE has been published recently [60], the older version possibly underestimating the risk of dying from CVD within 10 years. However, the version used in the present study was adapted to the Swiss population which allows a more accurate estimation of the risk in our specific cohort. Finally, obstructive sleep apnea is highly associated with cardiometabolic disturbances [61], but this information was available for only 8 inpatients and the variable could not, therefore, be adjusted for. Nevertheless, it can be mentioned that insomnia disorders are also associated with high BMI in the absence of obstructive sleep apnea [62, 63]. In addition, excluding patients suffering from obstructive sleep apnea did not change our results (data not shown).

## Conclusion

The present study involving a large cohort of psychiatric patients treated with weight-gain-inducing psychotropic drugs shows clinically significant associations between insomnia disorders and various metabolic disturbances including obesity, central obesity, hypertension, hyperglycemia, metabolic syndrome and risk of death from CVD within 10 years. Future studies should demonstrate whether better characterization and management of insomnia disorders would reduce the deterioration of cardiometabolic parameters in such psychiatric population at high risk of metabolic disturbances or if an improvement in metabolic health would improve sleep quantity and/or quality. 

## Supplementary Information

**Additional file 1:** **Supplementary Figure 1. **Procedure for participants’ inclusion in the study. **Supplementary Table 1.** Psychotropicmedications categorized by risk of weight gain. **Supplementary Table 2. **Sedative drugs used to determine insomnia disorders. **Supplementary Table 3.** Definition of metabolic disturbances and metabolicsyndrome [International Diabetes Federation (IDF) definition]. **Supplementary Table 4. **Association between insomnia disorders and cardiometabolic disturbances. **Supplementary Table5. **The main effect of insomnia disorders on cardiometabolic parameters adjustedfor time

## Data Availability

The datasets supporting the conclusions of this article were obtained from PsyMetab study. Requests to access the datasets should be directed to: research.psymetab@chuv.ch.

## Abbreviations

BMI: Body mass Index
BP: Blood pressure
CI: Confidence interval
CVD: Cardiovascular diseases
FPG: Fasting plasma glucose
FRS: Framingham Risk Score
HDL-C: High-density lipoprotein
ICD: International Classification of Diseases
IDF: International Diabetes Federation
LDL-C: Low-density lipoprotein
MetS: Metabolic syndrome
OR: Odds ratio
SCORE: Systematic Coronary Risk Estimation
SD: Standard deviation
TG: Triglycerides
Total-C: Total cholesterol
WC: Waist circumference
