# Publisher Correction: Insomnia disorders are associated with increased cardiometabolic disturbances and death risks from cardiovascular diseases in psychiatric patients treated with weight-gain-inducing psychotropic drugs: results from a Swiss cohort

**DOI:** 10.1186/s12888-022-04040-9

**Published:** 2022-07-08

**Authors:** Nermine Laaboub, Céline Dubath, Setareh Ranjbar, Guibet Sibailly, Claire Grosu, Marianna Piras, Didier Délessert, Hélène Richard-Lepouriel, Nicolas Ansermot, Severine Crettol, Frederik Vandenberghe, Carole Grandjean, Aurélie Delacrétaz, Franziska Gamma, Kerstin Jessica Plessen, Armin von Gunten, Philippe Conus, Chin B. Eap

**Affiliations:** 1 Unit of Pharmacogenetics and Clinical Psychopharmacology, Department of Psychiatry, Centre for Psychiatric Neuroscience, Lausanne University Hospital, University of Lausanne, 1008 Prilly, Prilly, Switzerland; 2 Center for Psychiatric Epidemiology and Psychopathology, Department of Psychiatry, Lausanne University Hospital, University of Lausanne, Prilly, Switzerland; 3 Prison Medicine and Psychiatry Service, Department of Psychiatry, Lausanne University Hospital, University of Lausanne, Prilly, Switzerland; 4 Unit of Mood Disorders, Department of Psychiatry, Geneva University Hospital, Geneva, Switzerland; 5 Les Toises Psychiatry and Psychotherapy Center, Lausanne, Switzerland; 6 Service of Child and Adolescent Psychiatry, Department of Psychiatry, Lausanne University Hospital, University of Lausanne, Prilly, Switzerland; 7 Service of Old Age Psychiatry, Department of Psychiatry, Lausanne University Hospital, University of Lausanne, Prilly, Switzerland; 8 Service of General Psychiatry, Department of Psychiatry, Lausanne University Hospital, University of Lausanne, Prilly, Switzerland; 9 School of Pharmaceutical Sciences, University of Geneva, University of Lausanne, Geneva, Switzerland; 10 Center for Research and Innovation in Clinical Pharmaceutical Sciences, University of Lausanne, Lausanne, Switzerland; 11 Institute of Pharmaceutical Sciences of Western Switzerland, University of Geneva, University of Lausanne, Lausanne, Switzerland

**Publisher Correction: BMC Psychiatry 22, 342 (2022)**

**https://doi.org/10.1186/s12888-022-03983-3**

Following the publication of the original article [1], the authors identified errors in the figure captions.

**Fig. 1** ^a^Defined using the International Diabetes Federation definition; ^b^BMI by 10 kg.m-^2^. ^c^ Estimated risk of death from cardiovascular diseases within 10 years using the Systematic Coronary Risk Estimation. Models were adjusted for age, sex, smoking status, and psychotropic medication (classified by the risk of weight gain), except the model for CVD which was adjusted only for psychotropic medication. ^1^ Models fitted with random effect at observation level. ^2^ Models fitted with random effect at patient level. ***: *p*-value < 0.001; **: *p*-value ≤ 0.01; *: *p*-value ≤ 0.05. Correction for multiple testing was applied using false discovery rate. Abbreviations: BMI body mass index, CVD cardiovascular diseases, HDL high-density lipoprotein, MetS metabolic syndrome, N number 
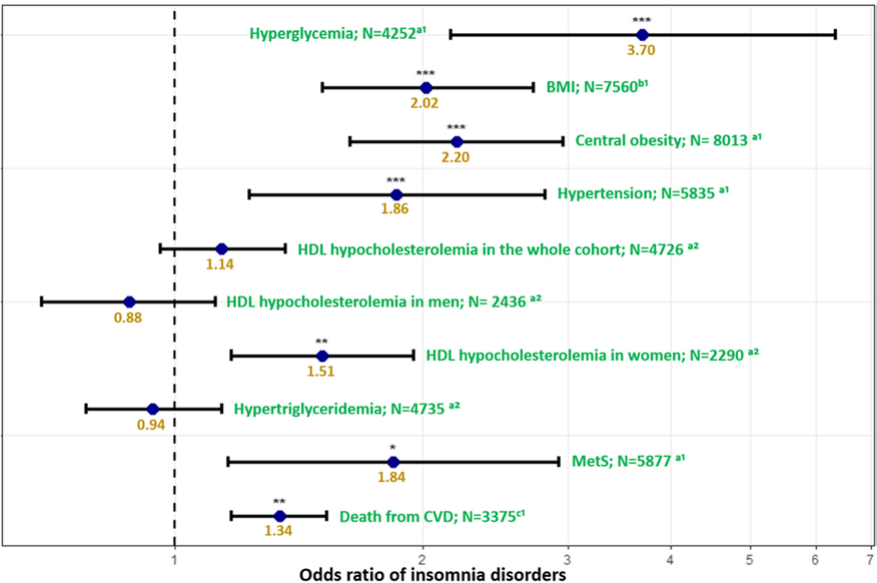


**Fig. 2** Model for FPG was adjusted for time, age, sex, smoking status and psychotropic medication. Model for TG was adjusted for time, age, interaction between age and insomnia disorders, sex, smoking status, setting of care (in/outpatient) and psychotropic medication. Model for HDL-C was adjusted for time, age, interaction between age and insomnia disorders, smoking status and psychotropic medication. Model for LDL-C was adjusted for time, age, interaction between age and insomnia disorders, sex and smoking status. Model for Total-C was adjusted for time, age, sex, interaction between age and insomnia disorders, sex, smoking status and psychotropic medication. Models for 10-year CVD risks (FRS and SCORE) were adjusted for time and psychotropic medication. ***:*p*-value < 0.001; **p-value < 0.01; *:*p*-value ≤ 0.05. Correction for multiple testing was applied using false discovery rate. Abbreviations: FPG fasting plasma glucose, FRS Framingham Risk Score, HDL-C high-density lipoprotein cholesterol, LDL-C low-density lipoprotein cholesterol, N number, SCORE  Systematic Coronary Risk Estimation, Total-C total cholesterol, TG triglycerides 
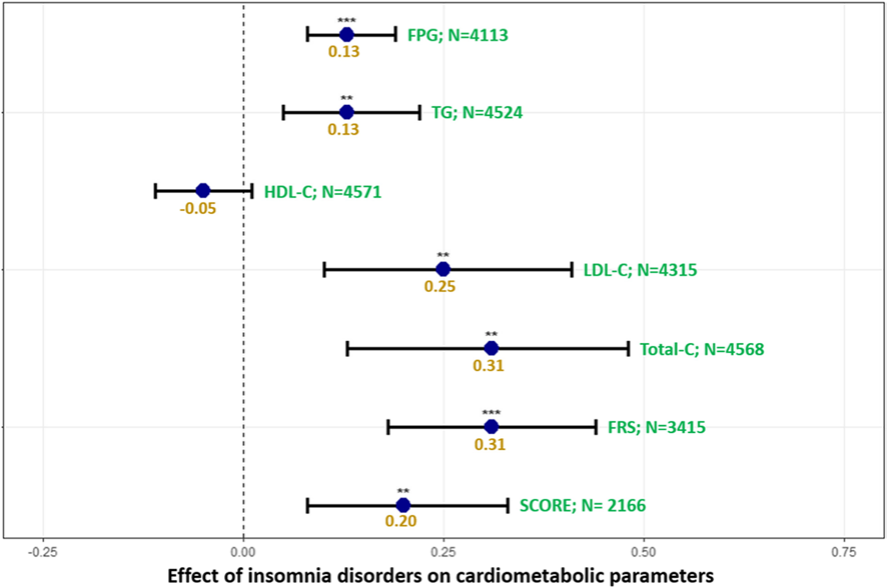


**Fig. 3** Models for BMI and waist circumference were adjusted for time, age, interaction between age and insomnia disorders, sex, smoking status, and psychotropic medication. Model for diastolic blood pressure was adjusted for time, age, interaction between age and insomnia disorders, sex and psychotropic medication. ***:*p*-value < 0.001; **: *p*-value < 0.01;*:*p*-value ≤ 0.05. Correction for multiple testing was applied using false discovery rate. Abbreviations: BMI body mass index, N number 
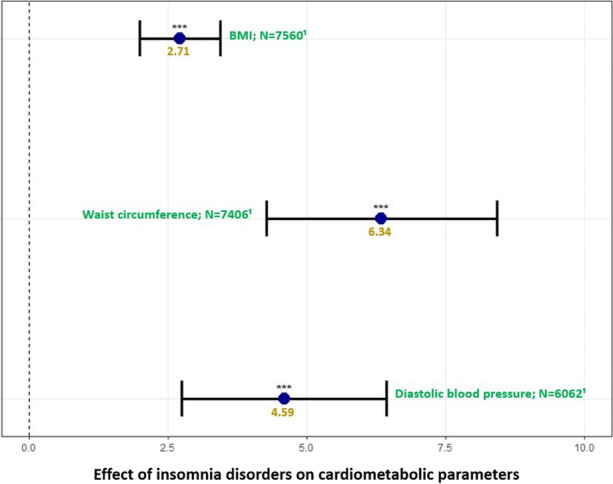


The original article [1] has been corrected.
