# Supplementary material for: Insomnia disorders are associated with increased cardiometabolic disturbances and death risks from cardiovascular diseases in psychiatric patients treated with weight-gain-inducing psychotropic drugs: results from a Swiss cohort
Source: BMC Psychiatry. 2022 May 17;22:342. doi: 10.1186/s12888-022-03983-3 (PMC9116036; doi:10.1186/s12888-022-03983-3)
Supplement: Supplementary file 1 — Additional file 1: Supplementary Figure 1. Procedure for participants’ inclusion in the study. Supplementary Table 1. Psychotropicmedications categorized by risk of weight gain. Supplementary Table 2. Sedative drugs used to determine insomnia disorders. Supplementary Table 3. Definition of metabolic disturbances and metabolicsyndrome [International Diabetes Federation (IDF) definition]. Supplementary Table 4. Association between insomnia disorders and cardiometabolic disturbances. Supplementary Table5. The main effect of insomnia disorders on cardiometabolic parameters adjustedfor time [file 12888_2022_3983_MOESM1_ESM.docx]

**Insomnia disorders and cardiometabolic disturbances in psychiatric patients taking weight-gain-inducing psychotropic drugs: results from a Swiss cohort: Supplementary files**

**Supplementary Table 1:** Psychotropic medications categorized by risk of weight gain

| **High risk** | **Medium risk** | **Low risk** |
| --- | --- | --- |
| Clozapine | Asenapine | Amisulpride |
| Olanzapine | Carbamazepine | Aripiprazole |
| Valproate | Amitriptyline | Brexpiprazole |
|  | Clomipramine | Chlorprothixene |
|  | Levomepromazine | Flupentixol |
|  | Lithium | Haloperidol |
|  | Mirtazapine | Lurasidone |
|  | Quetiapine | Sulpiride |
|  | Risperidone/Paliperidone | Tiapride |
|  | Trimipramine |  |
|  | Zuclopenthixol |  |

**Supplementary Table 2:** Sedative drugs used to determine insomnia disorders

| **Drugs** | **Dosages** | **Conditions** |
| --- | --- | --- |
| **Hypnotic, Z-drugs¹** | | |
| Zolpidem |  |  |
| Zopiclone |  |  |
| **Herbal sedatives¹** | | |
| Hops |  |  |
| Valerian |  |  |
| **Antidepressants** | | |
| Agomelatine | 25-50mg/day |  |
| Mianserin | 30-90mg/day | Indication confirmed in medical files or discharge letter. |
| Mirtazapine | 15mg/day | Indication confirmed in medical files or discharge letter. |
| Trazodone²^,^³ | 25-100mg/day | Indication confirmed in medical files or discharge letter.  If over 65 years old, indication confirmed in medical files |
| **Benzodiazepines** | | |
| Alprazolam |  | Indication confirmed in medical files or discharge letter. |
| Bromazepam |  | Indication confirmed in medical files or discharge letter. |
| Clorazepate |  | Indication confirmed in medical files or discharge letter. |
| Diazepam |  | Indication confirmed in medical files or discharge letter. |
| Flunitrazepam |  | Indication confirmed in medical files or discharge letter. |
| Flurazepam | 15-30mg/day | Indication confirmed in the medical files |
| Lorazepam |  | Indication confirmed in medical files or discharge letter. |
| Midazolam | 15-30mg/day | Indication confirmed in medical files or discharge letter. |
| Oxazepam | 15-30mg/day | Indication confirmed in medical files or discharge letter. |
| Triazolam |  | Indication confirmed in medical files or discharge letter. |
| **Antipsychotics** | | |
| Clotiapine | 20-40mg/day | Indication confirmed in medical files or discharge letter. |
| Levomepromazine | 25-50mg/day | Indication confirmed in medical files or discharge letter. |
| Quetiapine^²,⁴^ | 25-50mg/day | Indication confirmed in medical files or discharge letter except for 41 patients which were confirmed by quetiapine low dosage after 8PM.  If over 65 years old, indication confirmed in the clinical report |
| **Other drugs** | | |
| Clomethiazole |  | Only if administered after 8PM or indication confirmed in medical files |
| Chloral Hydrate | 500-1000mg/day | Only if administered after 8PM  or indication confirmed in medical files |
| Diphenhydramine | 25-50mg/day | Only if administered after 8PM  or indication confirmed in medical files |
| Doxylamine | 10-50mg/day | Only if administered after 8PM  or indication confirmed in medical files |
| Hydroxyzine | 25-50mg/day | Only if administered after 8PM  or indication confirmed in medical files |
| Melatonin**¹** |  |  |

¹ Medication used to define insomnia disorders in outpatients

^2^ In elderly patients, trazodone and quetiapine can be prescribed at low doses for other medical conditions; these indications were all confirmed by discharge letter or prescription condition in medical files.

^3^ Trazodone can be prescribed at 100mg/day for other medical reason, insomnia disorder status was confirmed by discharge letter.

⁴ 41 inpatients were considered to have insomnia disorders based only on quetiapine low dosage.

Abbreviation: Mg=milligrams

**Supplementary Table 3:** Definition of metabolic disturbances and metabolic syndrome [International Diabetes Federation (IDF) definition]:

|  |  |
| --- | --- |
| BMI | Dividing the body weight in kilograms by the square of the height in meters. |
| A. Central obesity | If waist circumference ≥ 94 cm (men) or 80 cm (women) or BMI > 30 kg/m². |
| B. Hypertension | If systolic BP ≥ 130 or diastolic BP ≥ 85 mmHg and/or specific treatment for blood pressure abnormality including diuretics, beta-blockers, renin-angiotensin inhibitors and/or calcium channel blockers. |
| C. Hyperglycemia | If fasting plasma glucose (FPG) ≥ 100 mg/dL (5.6 mmol/L) and/or specific treatment for type 2 diabetes including oral antidiabetics or insulin. |
| D. Hypertriglyceridemia | If serum triglycerides ≥ 150 mg/dL (1.7 mmol/L) and/or treatment for lipid abnormality including lipid-lowering agent (statins, fibrates). |
| E. HDL hypocholesterolemia | If HDL cholesterol < 40 mg/dL in men (1.03 mmol/L) or 50 mg/dL in women (1.29 mmol/L) and/or treatment for lipid abnormality including lipid-lowering agent (statins, fibrates). |
| Metabolic syndrome | If presence of A plus any two of B, C, D and E (IDF definition). |

Abbreviations: BMI=body mass index; BP=blood pressure; cm=centimeters; HDL=high density lipoprotein; Kg/m^2^=kilograms per square meter; mg/dL=milligram per deciliters; mmHG=millimeters of mercury; mmol/L=millimoles per liters.

**Supplementary Figure 1:** Procedure for participants’ inclusion in the study

15 844 observations

(4453 patients)

Excluding patients not from PsyMetab or PsyClin cohorts

13 037 observations

(3569 patients)

Excluding outpatients taking sedative medication(s)* except melatonin, zolpidem, zopiclone and herbal sedative

9345 observations

(2901 patients)

Excluding observations with missing values in BMI and waist circumference and blood pressure and plasma glucose and cholesterol levels.

**8954** observations

**(2861** patients)

*Because neither the doses nor the timing of the intake of sedative medication(s) were available for outpatients, insomnia disorders could not be ascertained and such patients were therefore excluded.

**Supplementary Table 4: Association between insomnia disorders and cardiometabolic disturbances**

|  | **Insomnia disorders** | | | | | | | | | | | |
| --- | --- | --- | --- | --- | --- | --- | --- | --- | --- | --- | --- | --- |
|  | Model 1 (N=7560)ᵃ | | | Model 2 (N=8013)ᵃ | | | Model 3 (N=4252)ᵃ | | | Model 4 (N=5835)ᵃ | | |
|  | **Odds ratio** | **95% CI** | **p-value** | **Odds ratio** | **95% CI** | **p-value** | **Odds ratio** | **95% CI** | **p-value** | **Odds ratio** | **95% CI** | **p-value** |
| **Age** (10 years) | 2.26 | 2.04 – 2.51 | **<10^-3^** | 2.19 | 1.98 – 2.43 | **<10^-3^** | 1.99 | 1.73 – 2.29 | **<10^-3^** | 2.15 | 1.91 – 2.43 | **<10^-3^** |
| **Sex** (Women) | 2.13 | 1.53 – 2.96 | **<10 ^-^**^3^ | 1.89 | 1.36 – 2.62 | **<10^-3^** | 1.98 | 1.28 – 3.08 | **0.002** | 2.79 | 1.93 – 4.04 | **<10 ^-^**^3^ |
| **Smokers** (yes) | 2.96 | 2.10 – 4.16 | **<10^-3^** | 3.01 | 2.16 – 4.20 | **<10^-3^** | 2.29 | 1.45 – 3.63 | **0.001** | 2.32 | 1.60 – 3.36 | **<10^-3^** |
| **Psychotropic medications¹**  Medium risk of WG  High risk of WG | 1.87  1.31 | 1.23 – 2.86  0.81 – 2.12 | **0.004**  0.27 | 1.82  1.55 | 1.20 – 2.75  0.95 – 2.50 | **0.005**  0.08 | 1.99  0.83 | 1.12 – 3.52  0.44 – 1.57 | **0.02**  0.56 | 1.19  2.38 | 0.71 – 1.98  1.27 – 4.45 | 0.49  **0.01** |
| **BMI** (10 kg/m^2^) | 2.02 | 1.51 – 2.72 | **<10^-3^** |  |  |  |  |  |  |  |  |  |
| **Central obesity**^2^ |  |  |  | 2.20 | 1.63 – 2.96 | **<10^-3^** |  |  |  |  |  |  |
| **Hyperglycemia**^2^ |  |  |  |  |  |  | 3.70 | 2.16 – 6.33 | **<10 ^-^**^3^ |  |  |  |
| **Hypertension**^2^ |  |  |  |  |  |  |  |  |  | 1.86 | 1.23 – 2.81 | **0.006** |

ᵃ Generalized linear mixed-effects models characterized by penalizing quasi-likelihood fitted with random effect at observation level.

P-values that are statistically significant after correction for multiple testing using the false discovery rate (FDR) are shown in bold.

The number of observations differs between the models due to missing data.

¹Psychotropic drugs were classified according to the weight gain risk as follows: **low risk**: amisulpride, aripiprazole, chlorprothixene, flupentixol, haloperidol, lurasidone, sulpiride, tiapride, brexpiprazole; **medium risk**: asenapine, carbamazepine, amitriptyline, clomipramine, levomepromazine, lithium, mirtazapine, paliperidone, quetiapine, risperidone, trimipramine, zuclopenthixol; **high risk**: clozapine, olanzapine, valproate, and were compared to low risk of WG.

^2^ Defined using International Diabetes Federation (IDF) criteria.

Due to the small proportion of patients treated with high risk of weight gain antipsychotics (20%) compared to medium risk (60%), association with insomnia disorders was not always significant.

-Abbreviations: BMI=body mass index; CI=confidence interval; Kg/m^2^=kilograms per square meters; N=number; WG=weight-gain.

**Supplementary Table 4: Association between insomnia disorders and cardiometabolic disturbances** **(continued)**

|  | **Insomnia disorders** | | | | | | | | |
| --- | --- | --- | --- | --- | --- | --- | --- | --- | --- |
|  | Model 5 (N=2290)ᵇ | | | Model 6 (N=5877)ᵃ | | | Model 7 (N=3375)ᵃ | | |
|  | **Odds ratio** | **95% CI** | **p-value** | **Odds ratio** | **95% CI** | **p-value** | **Odds ratio** | **95% CI** | **p-value** |
| **Age** (10 years) | 1.49 | 1.37 – 1.64 | **<10^-3^** | 2.15 | 1.92 – 2.41 | **<10^-3^** |  |  |  |
| **Sex** (Women) |  |  |  | 2.09 | 1.45 – 3.01 | **<10^-3^** |  |  |  |
| **Smokers** (yes) | 1.38 | 1.03 – 1.85 | **0.03** | 2.59 | 1.78 – 3.79 | **<10^-3^** |  |  |  |
| **Psychotropic medications¹**  Medium risk of WG  High risk of WG | 1.11  1.29 | 0.80 – 1.54  0.87 – 1.92 | 0.54  0.20 | 2.10  1.54 | 1.31 – 3.37  0.90 – 2.64 | **0.003**  0.12 | 1.45  2.99 | 0.74 – 2.82  1.29 – 6.90 | 0.24  **0.02** |
| **HDL hypocholesterolemia in women**^2^ | 1.51 | 1.17 – 1.95 | **0.002** |  |  |  |  |  |  |
| **MetS**^2^ |  |  |  | 1.84 | 1.16 – 2.92 | **0.01** |  |  |  |
| **Death from CVD**^3^ |  |  |  |  |  |  | 1.34 | 1.17 – 1.53 | **0.001** |

ᵃ Generalized linear mixed effects models by penalizing quasi-likelihood fitted with random effect at observation level.

ᵇ Generalized linear mixed effects models by penalizing quasi-likelihood fitted with random effect at patient level.

P-values that are statistically significant after correction for multiple testing using the false discovery rate (FDR) are shown in bold.

The number of observations differs among models due to missing data.

¹Psychotropic drugs were classified according to risk of weight gain as follows: **low risk**: amisulpride, aripiprazole, chlorprothixene, flupentixol, haloperidol, lurasidone, sulpiride, tiapride, brexpiprazole; **medium risk**: asenapine, carbamazepine, amitriptyline, clomipramine, levomepromazine, lithium, mirtazapine, paliperidone, quetiapine, risperidone, trimipramine, zuclopenthixol; **high risk**: clozapine, olanzapine, valproate, and were compared to low risk of WG.

^2^Defined using the International Diabetes Federation (IDF) definition.

^3^Risk of death from CVD within 10 years, calculated using the Systematic Coronary Risk Estimation (SCORE).

Due to the small proportion of patients treated with antipsychotics with high risk of weight gain (20%) compared to medium risk (60%), association with insomnia disorders was not always significant.

-Abbreviations: CI=confidence interval; CVD=cardiovascular diseases; HDL=high-density lipoprotein; MetS=metabolic syndrome; N=number; WG=weight-gain.

**Supplementary Table 5: The main effect of insomnia disorders on cardiometabolic parameters adjusted for time**

|  | | Es | 95% CI | p-value |  | | Es | 95% CI | p-value |  | | Es | 95% CI | p-value |
| --- | --- | --- | --- | --- | --- | --- | --- | --- | --- | --- | --- | --- | --- | --- |
| Body mass index (N=7560)^1^ | Time | 0.18 | 0.06 – 0.31 | **0.003** | Waist circumference (N=7406)^2^ | Time | 0.91 | 0.57 – 1.25 | **<10^-3^** | Systolic blood pressure (N=6064)^3^ | Time | -0.29 | -0.70 – 0.12 | 0.16 |
|  | Insomnia disorders | 2.71 | 1.99 – 3.44 | **<10^-3^** |  | Insomnia disorders | 6.34 | 4.27 – 8.42 | **<10^-3^** |  | Insomnia disorders | 0.62 | -1.77 – 3.01 | 0.61 |
|  | Age | 0.06 | 0.05 – 0.07 | **<10^-3^** |  | Age | 0.30 | 0.27 – 0.33 | **<10^-3^** |  | Age | 0.34 | 0.31 – 0.37 | **<10^-3^** |
|  | Insomnia disorders * age | -0.06 | -0.07 – -0.04 | **<10^-3^** |  | Insomnia disorders * age | -0.12 | -0.17 – -0.08 | **<10^-3^** |  | Insomnia disorders * age | -0.05 | -0.10 – 0.01 | 0.08 |
| Diastolic blood pressure (N=6062) ^4^ | Time | 0.09 | -0.23 – 0.40 | 0.59 | Fasting plasma glucose (N=4113)^5^ | Time | 0.06 | 0.03 – 0.08 | **<10^-3^** | Triglycerides (N=4473)^6^ | Time | 0.00 | -0.02 – 0.01 | 0.80 |
|  | Insomnia disorders | 4.59 | 2.75 – 6.43 | **<10^-3^** |  | Insomnia disorders | 0.13 | 0.08 – 0.19 | **<10^-3^** |  | Insomnia disorders | 0.13 | 0.05 – 0.22 | **0.002** |
|  | Age | 0.20 | 0.17 – 0.22 | **<10^-3^** |  | Age | 0.012 | 0.01 – 0.013 | **<10^-3^** |  | Age | 0.01 | 0.008 – 0.013 | **<10^-3^** |
|  | Insomnia disorders * age | -0.12 | -0.16 – -0.08 | **<10^-3^** |  |  |  |  |  |  | Insomnia disorders * age | -0.006 | -0.009 – -0.003 | **<10^-3^** |

P-values that are statistically significant after correction for multiple testing using the false discovery rate (FDR) are shown in bold.

The number of observations differs among the models due to missing data.

Models 1, 2 and 5 were adjusted for sex, smoking status and psychotropic medication. Models 3 and 4 were adjusted for sex and psychotropic medication. Model 6 was adjusted for sex, smoking status, psychotropic medication and setting of care.

To ensure normal distribution, triglycerides was log-transformed and time was scaled to have a mean of 0 and SD of 1; therefore, the coefficients of these regressions should be interpreted as percentage changes.

-Abbreviations: CI=confidence interval; Es=estimate; N=number

**Supplementary Table 5: The main effect of insomnia disorders on cardiometabolic parameters adjusting for time (continued)**

|  | | Es | 95% CI | p-value |  | | Es | 95% CI | p-value |  | | Es | 95% CI | p-value |
| --- | --- | --- | --- | --- | --- | --- | --- | --- | --- | --- | --- | --- | --- | --- |
| HDL cholesterol (N=4571)^7^ | Time | -0.01 | -0.02 – 0.00 | 0.26 | HDL cholesterol in women (N=2226)^8^ | Time | -0.02 | -0.04 - -0.01 | **0.009** | LDL cholesterol (N=4315)^9^ | Time | 0.02 | -0.002 – 0.052 | 0.07 |
|  | Insomnia disorders | -0.05 | -0.11 – 0.01 | 0.13 |  | Insomnia disorders | -0.06 | -0.16 - -0.04 | 0.26 |  | Insomnia disorders | 0.25 | 0.10 – 0.40 | **0.001** |
|  | Age | 0.0005 | -0.0001 – 0.002 | 0.12 |  | Age | 0.001 | 0.0003– 0.003 | 0.01 |  | Age | 0.015 | 0.013 – 0.017 | **<10^-3^** |
|  | Insomnia disorders * age | 0.0001 | -0.0005 – 0.002 | 0.23 |  | Insomnia disorders * age | 0.002 | -0.001 – 0.002 | 0.52 |  | Insomnia disorders * age | -0.006 | -0.010 – 0.003 | **<10^-3^** |
| Total cholesterol (N=4568) ^10^ | Time | 0.01 | -0.02 – 0.04 | 0.56 | FRS (N=3415)^11^ | Time | -0.06 | -0.12 – -0.00 | 0.06 | SCORE (N=2166)^12^ | Time | -0.15 | -0.21 – -0.08 | **<10^-3^** |
|  | Insomnia disorders | 0.31 | 0.13 – 0.48 | **0.001** |  | Insomnia disorders | 0.31 | 0.18 – 0.44 | **<10^-3^** |  | Insomnia disorders | 0.20 | 0.08 – 0.33 | **0.002** |
|  | Age | 0.021 | 0.018 – 0.023 | **<10^-3^** |  |  |  |  |  |  |  |  |  |  |
|  | Insomnia disorders * age | -0.009 | -0.013 - -0.005 | **<10^-3^** |  |  |  |  |  |  |  |  |  |  |

P-values that are statistically significant after correction for multiple testing using the false discovery rate (FDR) are shown in bold.

The number of observations differs among the models due to missing data.

Model 7 and 10 were adjusted for sex, smoking status and psychotropic medication. Model 8 was adjusted for smoking status and psychotropic medication. Model 9 was adjusted for sex and smoking status. Models 11 and 12 were adjusted for psychotropic medication.

To ensure normal distribution, FRS and SCORE (10-year CVD risks) were log-transformed and time was scaled to have a mean of 0 and SD of 1, therefore, the coefficients of these regressions should be interpreted as percentage changes.

-Abbreviations: CI=confidence interval; Es= estimate; FRS=Framingham risk score; HDL=high density lipoprotein; LDL=low density lipoprotein; N=number; SCORE=Systematic Coronary Risk Estimation.
